# *Cryptosporidium* species diagnosis in handlers of domestic pigeons in Baghdad City: Molecular and microscopic approaches

**DOI:** 10.5455/javar.2023.j730

**Published:** 2023-12-31

**Authors:** Yahya F. Hashim, Mohammed Th. S. Al-Zubaidi

**Affiliations:** Department of Parasitology, College of Veterinary Medicine, University of Baghdad, Baghdad, Iraq

**Keywords:** Baghdad city, *Cryptosporidium*, domestic pigeons, handlers, nested PCR

## Abstract

**Objective:**

The aim of this study is to detect the prevalence and diagnosis of molecular characteristics of Cryptosporidium infection in handlers of domestic pigeons in Baghdad City. Traditional and molecular diagnostic methods were employed to detect and identify Cryptosporidium species.

**Materials and methods:**

Sixty stool samples were collected from the handlers of domestic pigeons, and various techniques, including direct smear, flotation concentration, staining methods, and DNA extraction coupled with nested polymerase chain reaction (PCR), were utilized.

**Results:**

The results obtained from traditional methods indicated an overall infection rate of 55% in handlers of domestic pigeons, while significant variations were noted between male and female handlers. Age group 21–40 years were found to have higher infection rates were found to have higher infection rates. The prevalence of Cryptosporidium also displayed temporal variability throughout the study period. Molecular analysis using nested PCR revealed higher infection rates of 86% in handlers of domestic pigeon samples. Genotyping and phylogenetic analysis identified the presence of Cryptosporidium parvam species in handlers of domestic pigeons, indicating zoonotic transmission potential.

**Conclusion:**

These findings underscore the high prevalence of Cryptosporidium infection among handlers of domestic pigeons in Baghdad City.

## Introduction

Cryptosporidium species are harmful parasites that are frequently detected in the gastrointestinal tracts of several host families [1]. The earliest description of this phenomenon was provided by Tyzzer in 1907, as shown in a study conducted on mice [1]. The first documented cases of human infection in both children and adults were reported in 1976. The transmission of Cryptosporidium infection in humans and domestic pigeons is a matter of public health concern, according to a study by [1]. Birds are infected with Cryptosporidium spp., and the species Cryptosporidium meleagridis is frequently associated with cases of human cryptosporidiosis [2]. This is mostly due to its propensity to be transmitted through many routes, such as food, water, and direct contact with infected animals [3]. Pigeons serve as carriers and have the potential to transmit infections to both other avian species and those that handle them, therefore highlighting their significance in the realm of zoonotic transmission. Cryptosporidium infection is a highly significant disease that induces intestinal infection in people, animals, and avian species. Cryptosporidium species can invade several anatomical areas within the host’s body, including the gut, stomach, and respiratory system [4].

The prevalence of Cryptosporidium infection among domestic pigeon handlers in Iraq remains underexplored, with limited research on this topic making it difficult to assess the extent of transmission within this specific population [5]. Understanding the prevalence and transmission dynamics of Cryptosporidium in domestic pigeon handlers is crucial for implementing effective control measures and reducing the burden of infection [6]. Therefore, the study objective was the detection of Cryptosporidium spp. in handlers of domestic pigeons inside Baghdad City using both conventional methods and polymerase chain reaction (PCR) techniques.

## Materials and Methods

### Ethical approval

This study was approved by the rules set by the Baghdad University of Veterinary Medicine and the Committee of the Iraqi Ministry of Health, al-Karkh Health Directorate with No. 31.

### Sample collection

Samples of fresh stool were collected from 60 pigeon handlers, both male and female, of varying ages, collected from peripheral health care centers (PHCs) within Baghdad city. The collection period lasted for six months, starting in October 2022 and ending in March 2023.

### Sample preparation

Samples were collected from patients of PHCs located in Baghdad who suffered from non-bloody diarrhea and handled domestic pigeons. All samples were kept in a cold box, sealed in disposable, sterile containers with labels [7], and then moved to the University of Baghdad College of Veterinary Parasitology Lab. Samples were split into two parts. The initial portion of each sample was examined for parasites and confirmed by a modified Ziehl–Neelson stain [8]. A microscope with an oil immersion lens (×100) was used to detect protozoan oocysts on stained slides. The latter portion of the molecular method was stored at −20°C for testing in the veterinary college’s internal and preventive medicine department.

### Genomic DNA estimation

Genomic DNA was extracted from all 80 stool samples using the Geneaid DNA extraction kit in Taiwan, and it was done according to company instructions. The quantification of the genomic DNA was performed using a nanodrop spectrophotometer (THERMO, USA), which measures absorbance at 260/280 nm and the concentration of the extracted DNA, which ranged from 5 to 50 ng/µl.

### Primers

The PCR primers designed for Cryptosporidium detection were crafted using National Center for Biotechnology Information (NCBI)-Genbank and primer 3 plus (AF442484.1), sourced from Microgen/Korea. This study employed two sets of PCR primer pairs. The initial set, comprising cycle of primer (CPr) I (5′-GGG TAT TGG CCT ACC GTG G-3′) and CPr II (5′-ACC GGA TCA TTC AAT CGG TAG G-3′), was utilized in the first PCR round, capturing a segment of 1,320 base pairs within the small ribosomal subunit of Cryptosporidium. The second primer set, CPr III (5′-GGG GAT CGA AGA CGA TCA G-3′) and CPr IV (5′-CCA TTT CCT TCG AAA CAG GA-3′) was employed in the subsequent round of PCR, specifically targeting a region of 529 base pairs within the amplified ribosomal locus.

### PCR reaction preparatory component

The PCR process utilized GoTaq Green PCR Master Mix (Promega, USA). In the initial round, a mixture of 12.5 µl of the master mix, 5 µl of DNA template, 2 µl each of 18SrRNA forward and reverse primers (10 pmol each), and 3.5 µl of PCR water was prepared. In the subsequent round, 12.5 µl of the master mix, 1 µl of PCR product DNA, 1 µl each of 18SrRNA forward and reverse primers (10 pmol each), and 9.5 µl from the water of PCR were used. The PCR tubes were then subjected to centrifugation at 3,000 rpm for 3 min. The cycling included the beginning of denaturation at 95°C for 5 min, followed by 35 cycles of 30 sec at 95°C, 30 sec at 55°C, and 2 min at 72°C. A final extension step at 72°C for 5 min was followed by a holding phase at 4°C. The success of the amplification process was confirmed on a 1.5% electrophoresis agarose gel stained with ethidium bromide in 1× Tris Borate EDTA buffer.

**Figure 1.**
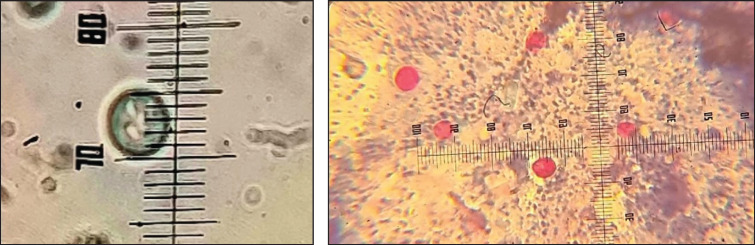
(A) A direct examination of *Cryptosporidium* oocysts at magnification of 100×. (B) An examination of *Cryptosporidium* oocysts by Modified Ziehl-Neelsen stain power at magnification of 100×.

### DNA sequencing

The PCR products were sequenced at Macrogen Inc. in South Korea. To ensure accuracy, the obtained sequences were compared against the Cryptosporidium rRNA reference databases from NCBI using BioEdit Sequence Alignment Editor Software Version 7.1 (DNA STAR, Madison, WI). SnapGene Viewer Version 4.0.4 was then used to visualize chromatograms illustrating any sequence variations.

### Phylogenetic analysis

In this study, a Cryptosporidium tree was created using Clustal Omega. The complete inclusive tree, which incorporates the variants that were observed, was shown as a polar cladogram utilizing the interactive tree of life tool.

### Statistical analysis

Using the chi-square test, SPSS Version 23.0 for Windows was used to validate the connection between this parasite and each of the researched factors. Microsoft Excel 356 built the variable database to be significant at *p* ≤ 0.05 judged were variances.

## Results and Discussion

The findings show a significant prevalence of Cryptosporidium infection among handlers of domestic pigeons in Baghdad city. Out of the 60 stool samples examined between October 1st, 2022, and the end of March 2023, a notable 55% (33/60) tested positive for Cryptosporidium spp. This finding is consistent with a study conducted in Al Najaf [9], which reported a similar infection rate of 58%, providing further support for these results.

In Mosul city, the observed infection rate of 43.56% (34/78) [10] aligns with findings, indicating a relatively high prevalence of Cryptosporidium infection among handlers of domestic pigeons in urban areas. However, the results sharply contrast with those from studies conducted elsewhere in Iraq, which reported significantly lower infection rates of 14% (14/100) and 29% (32/110) [11,12] subsequently. These discrepancies may be attributed to regional variations in hygiene practices, environmental conditions, or other contributing factors. Under lenses, Cryptosporidium spp. oocysts showing oval by traditional techniques and having a red circular body, blue backdrop, and apparent hallow by stain of modified Zehl–Neelson results are shown in Figure 1.

### *Infection rate of* Cryptosporidium *according to gender*

Handlers’ total rate of infection in males was 30 (55.55%) out of 54 males examined, while in females, it was 3 (50%) out of six females examined. Significant disparities exist between the sexes (*p* < 0.05), as shown in Table 1.

Comparing these results with a study carried out by Al-Yasary and Faraj [13] in Karbala Province, some interesting disparities emerge, but there is no significant difference between genders. Specifically, the male percentage was 27.41% (17/62), while the female percentage was 23.68% (9/38). This discrepancy highlights potential regional variations in the relationship between gender and Cryptosporidium susceptibility. It is important to note that these differing findings may be attributed to a variety of factors, including regional variations in hygiene practices, environmental conditions, or other local influences.

### *Infection rate of* Cryptosporidium *according to age groups*

The analysis of Cryptosporidium infection rates across different age groups among handlers reveals intriguing patterns. Notably, the highest rates were found in individuals between the ages of 21 and 40, with 62.9% (17/27) testing positive for the parasite. This trend continues, though slightly attenuated, in the age group of 41–59 years, where 55.56% (10/18) were found to be infected. Among individuals younger than 20 years, a relatively lower but still notable infection rate of 41.67% (5/12) was recorded. In contrast, the age group of 60 years and older exhibited the smallest infection rate at 33.33% (1/3) out of the handlers examined.

These findings demonstrate a clear association between age and the susceptibility to Cryptosporidium infection, with younger individuals and those in middle adulthood exhibiting higher rates of infection, with significant differences (*p* < 0.05) shown in Table 2.

**Table 1.** Infection rate of *Cryptosporidium* in handlers according to sex.

Sex	No. of examined	No. of infected	Percentage
Males	54	30	55.55
Females	6	3	50.00
Total	60	33	55.00
*p*-value	0.0001 *		

* = With significant difference (*p* < 0.05)

**Table 2.** Infection rate of *Cryptosporidium* in handlers according to age groups.

Age group (Year)	No. of examined	No. of infected	Percentage
<20	12	5	41.67
21–40	27	17	62.96
41–59	18	10	55.5
≥60	3	1	33.33
Total	60	33	55.00
*p*-value			0.0001 **

** = With significant difference (*p* < 0.05)

The findings are in line with other investigations conducted in Kirkuk province [14] and Al Diwania province [11], which also reported higher statistically significant rates of Cryptosporidium infections among individuals within the age range of 21–40 years. This concordance suggests a consistent pattern across different regions, emphasizing the heightened vulnerability of individuals in this age bracket.

Interestingly, the findings contradict those of the Iraqi study conducted by Sayal [9] in Najf province and [10] in Mosul province, which reported no significant variations in Cryptosporidium infection rates based on age groups. This discrepancy may stem from regional disparities in hygiene practices, environmental conditions, or other contributing factors that warrant further investigation. Furthermore, a study conducted in Karbala province highlighted the significance of the age group under 20, where a noteworthy infection rate of 23/75 (approximately 30.67%) was observed [13]. This underscores the importance of considering age-related factors when assessing Cryptosporidium prevalence, as different age groups may exhibit distinct susceptibilities to infection. Overall, these findings provide valuable insights for targeted preventive measures and interventions.

**Table 3.** Infection rate of *Cryptosporidium* in handlers according to months.

Month	No. Of examined handlers	No. Of infected handlers	Percentage
October 2022	10	3	30.00
November	10	2	20.00
December	10	8	80.00
January 2023	10	9	90.00
February	10	9	90.00
March	10	2	20.00
Total	60	33	55.00
*p*-value			0.0001 **

** = With significant difference (*p* < 0.05)

### *Rate of infection with* Cryptosporidium *spp. by month*

The temporal analysis of Cryptosporidium spp. infection rates among pigeon handlers throughout the study period reveal noteworthy seasonal variations. The prevalence of Cryptosporidium was consistently observed across all months. However, the infection rates exhibited distinctive patterns, with significant differences (*p* ≤ 0.05) between the rates of infection in Table 3.

During February and January, the infection rates peaked at 90% (9/10), indicating a substantial prevalence of the parasite during these winter months. In December, the infection rate remained notably high, at 80% (8/10). In contrast, November and March exhibited considerably lower infection rates of 20% (2/10) each. These fluctuations in infection rates demonstrate a clear seasonal influence on Cryptosporidium transmission dynamics among pigeon handlers.

This study supports previous research indicating that seasonal factors affect Cryptosporidium prevalence. For instance, Sayal [9] reported a 24.1% infection rate in humans during February, while AL-Yasary and Faraj [13] noted a significantly higher rate of 46.66% in the same month. Additionally, Yaqoob et al. [15] identified January as having the highest infection rate at 36.58%. These consistent trends further support the hypothesis that the winter months, particularly January and February, are associated with elevated Cryptosporidium transmission. In contrast, Al-Jawasim and Al-Khaled [16] reported a steady 30% infection rate among handlers in September (autumn), suggesting a different seasonal pattern in the study region. Similarly, Hadi and Faraj [17] observed peak infection rates in March and April, reaching 35%. These discrepancies emphasize the regional variability in Cryptosporidium transmission dynamics and highlight the importance of considering local environmental conditions and human behaviors when interpreting seasonal trends.

**Figure 2.**
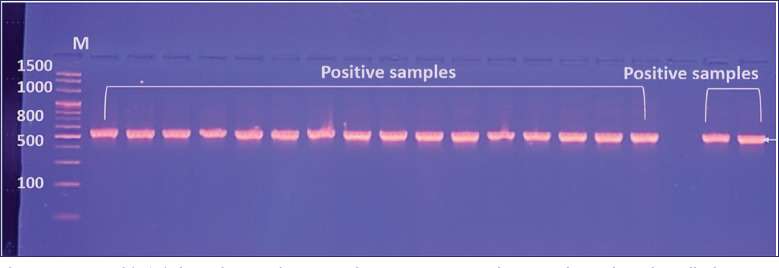
Agarose gel (1.5%) electrophoreses of *Cryptosporidium* spp. genomic DNA the PCR product analysis of a small subunit ribosomal RNA gene in *Cryptosporidium* spp. from a Domistic pigeon drop sample. Where M: marker (1500–100 bp), lanes showed some positive *Cryptosporidium* spp. at (529 bp) PCR product.

The seasonal variations in Cryptosporidium infection rates require targeted surveillance and prevention, especially in the winter. Public health interventions to reduce Cryptosporidium infection in pigeons and their handlers must understand seasonal transmission dynamics.

### Handlers of domestic pigeons: sequence analysis

The samples, collected randomly from 60 handlers of domestic pigeons in Baghdad city, on agarose gel (1.5%) have a unique 529 bp, as shown in Figure 2. DNA sequences in this study match those in the homology sequence identity study of local Cryptosporidium spp. IQB-Human No.1-No.5 isolates and NCBI-Genbank-related Cryptosporidium parvum (DQ898159.1) closely related to the Chinese isolate had genetic homology sequence identities of 99.64%–99.84%, either in the gene bank under accession numbers (OR123514, OR123515, OR123516, OR123517, OR123518), as shown in Table 4.

**Table 4.** NCBI-BLAST homology sequence identity percentage between *Cryptosporidium* spp. Human isolates and NCBI-BLAST closed genetic-related *Cryptosporidium* species isolate.

*Cryptosporidium* spp. Isolate No.	Accession number	Homology sequence identity (%)			
		Identical *Cryptosporidium* spp.	Accession number	Country	Identity (%)
**IQB-Human No.1**	**OR123514**	*C. parvum*	**DQ898159**	**China**	**99.65**
**IQB-Human No.2**	**OR123515**	*C. parvum*	**DQ898159**	**China**	**99.84**
**IQB-Human No.3**	**OR123516**	*C. parvum*	**DQ898159**	**China**	**99.64**
**IQB-Human No.4**	**OR123517**	*C. parvum*	**DQ898159**	**China**	**99.84**
**IQB-Human No.5**	**OR123518**	*C. parvum*	**DQ898159**	**China**	**99.84**

**Figure 3.**
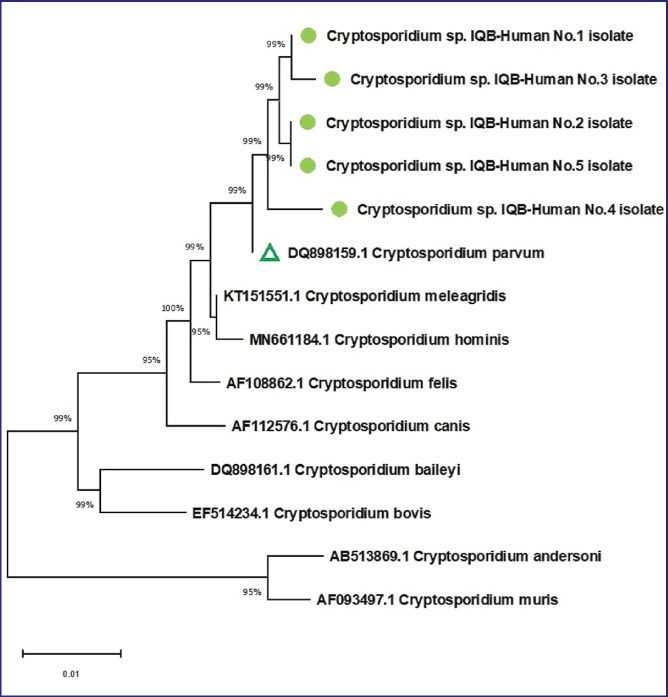
Phylogenetic tree study of *18SrRNA* gene partial sequences in local *Cryptosporidium* species from humans for genetic species typing.

### Handlers of domestic pigeons phylogenetic analysis

The phylogenetic tree analysis further elucidated the genetic relationships, demonstrating a close affinity between the Cryptosporidium spp. IQB-Human No.1-No.5 isolate and the NCBI-BLAST C. parvum (DQ898159.1). The total genetic changes observed were a mere 0.01%, confirming the genetic similarity between these isolates, as illustrated in Figure 3.

The genetic data presented in this study resonate with previous research. For instance, a study in Bangladesh reported a similar trend, albeit at a lower percentage, with *C*. parvum found in 1.0% (2/197) of cases [18]. Similarly, in Egypt, a study of handlers identified *C*. parvum in 6% (3/50) of pigeon handlers [19]. Notably, a study in Al-Kut and Al-Zahraa Teaching Hospital in Wasit province found a high prevalence, with 60% (48/80) of patients diagnosed with *C*. parvum being infected [20]. It is essential to acknowledge that *C*. parvum has been detected in various species, posing a potential health risk to the general public, including pigeons, turkeys, broiler flocks, and layer flocks [21,22].

According to this study, these populations’ unsanitary habits and shared environments increase risk. Pigeons, other animals, and humans need proper hygiene and prevention to avoid Cryptosporidium spp. These findings inform Cryptosporidium transmission dynamics and public health interventions.

## Conclusion

These findings underscore the high prevalence of Cryptosporidium infection among handlers of domestic pigeons in Baghdad City. The study emphasizes the need for further epidemiological investigations and understanding of Cryptosporidium transmission dynamics in these populations.

## List of abbreviations

PHCs, peripheral health care centers; nested PCR, nested polymerase chain reaction; CPr, cycle of primer; NCBI, National Center for Biotechnology Information.
